# The dual (activating/suppressive) effect of extracellular TatHIV-1 is driven by the infalmmatory microenvironment of infected lymphoid foci

**DOI:** 10.1186/1742-4690-9-S1-P35

**Published:** 2012-05-25

**Authors:** Hélène Le Buanec, Thomas Sené, Armand Bensussan, Robert Gallo, Daniel Zagury

**Affiliations:** 1 INSERM U976, F-75475, Paris, France; 2 Université Paris Diderot, Sorbonne Paris Cité, Laboratory of Immunology, Dermatology & Oncology, UMR-S 976, F-75475, Paris, France; 3 Service de dermatologie, Hopital Saint Louis, F-75010, Paris, France; 4 Institute of Human Virology, University of Maryland Baltimore, Maryland, USA; 5 Neovacs SA, Paris, France

## 

It has been shown that HIV-1 infects activated but not resting CD4+ T cells [1] and that CPE induced by viral replication together with the immunosuppressive effect triggered by extracellular Tat protein [2] account for the decrease of CD4+ T cell count in infected patients. In lymphoid foci, dependent on the level of viral infection, the stromal microenvironment surrounding immune cells could include, together with extracellular Tat [3] and circulating antiviral IFN-α, inflammatory innate factors such as ATP and derivatives released by CPE-derived dead cells.

We show that, according to its concentration and the presence of inflammatory factors (IFN-α, ATP and ATP-derivatives), Tat protein may exert either an activation with enhanced production of IL2 or an immune suppression of stimulated CD4+ T cells subpopulations.

The double-edged sword of Tat activity on CD4+ T cells could account for its immunopathogenic effects both at the early stage of infection (by allowing CD4+ T cells activation and viral replication) and at late stages (by inducing immuosuppression, source of opportunistic infections). Indications for targeting Tat protein by therapeutic vaccines in subgroups of HIV-1 infected patients will be discussed.
